# Supplementary material for: Leucine-Enriched Diet Reduces Fecal MPO but Does Not Protect Against DSS Colitis in a Mouse Model of Crohn’s Disease-like Ileitis
Source: Int J Mol Sci. 2024 Nov 1;25(21):11748. doi: 10.3390/ijms252111748 (PMC11545852; doi:10.3390/ijms252111748)
Supplement: Supplementary file 1 [file ijms-25-11748-s001.zip › ijms-3272888-supplementary.pdf]

Supplementary Table S1. Histology scores of different diet groups.

Group	AD+AA	AD	CH+AA	CH
% ulceration (0-3)	2.2	2.2	2.7	0.4
% re-epithelization (0-4)	0.9	0.7	1.0	0.1
Active inflammation. (0-3)	2.4	2.7	2.7	1.8
% cross sectional area (0-4)	2.6	2.0	2.7	1.2
Active inflammation index	6.2	5.7	7.0	2.2
Chronic inflammation (0-3)	2.0	1.7	2.0	1.5
% cross sectional area (0-4)	1.8	2.0	2.3	1.0
Chronic inflammation. index - colon	3.6	3.7	4.7	1.5
Transmural inflammation (0-3)	1.8	1.7	2.0	0.7
% cross section (0-4)	1.2	1.0	1.0	0.5
Transmural index	2.2	1.7	2.0	0.7
Total	15.1	13.8	17.3	4.8

Supplementary Table S2. Diet Composition

Diet	Standard CHOW (CH)	Leucine rich CHOW (CH+AA)	American Diet (AD)	Leucine rich AD (AD+AA)
**Ingredient (g)**	**g**	**g**	**g**	**g**
Casien	200	0	32	0
L-Leucine	0	14.33	0	28.1
L-Cystine	3	2.22	0.5	2
Beef, Powdered (5013)	0	0	94.5	0
Chicken, Powdered (ChickPRO)	0	0	56.1	0
Egg White, Dried	0	0	21	0
Fish Protein Isolate (Earth2Sea Nutrients)	0	0	26.2	0
Corn Starch	381	381	227.8	228.61
Dextrose	150	150	0	0
Maltodextrin 10	110	110	66	66.24
Lactose	0	0	35	35.12
Sucrose	9	8.5	139	138.96
Cellulose, BW200	75	75	0	0
Inulin	25	25	32	32
Soybean Oil	70	72.4	0	0
Butter, Anhydrous	0	0	30	40.96
Canola Oil	0	0	16	21.84
Corn Oil	0	0	36	49.15
Peanut Oil	0	0	8	10.92
Primex-Z (Shortening)	0	0	22	30.03
Mineral Mix S10026	10	10	10	10
DiCalcium Phosphate	13	13	13	13
Calcium Carbonate	5.5	5.5	5.5	5.5
Potassium Citrate, 1 H2O	16.5	16.5	16.5	16.5
Vitamin Mix V10001C	1	1.5	1	1.5
Choline Bitartrate	2	2	2	2
FD&C Yellow Dye #5	0.025	0.025	0.05	0
FD&C Red Dye #40	0	0.025	0	0
FD&C Blue Dye #1	0.025	0	0	0
**Protein (gm)**	**177**	**177**	**174**	**174**
Protein from Casein	174	0	27.8	27.8
Protein from Beef, Powdered (5013)	0	0	54.8	54.8

Diet	Standard CHOW (CH)	Leucine rich CHOW (CH+AA)	American Diet (AD)	Leucine rich AD (AD+AA)
Protein from ChickPRO	0	0	47.7	47.7
Protein from Egg White, Dried	0	0	17	17
Protein from Fish Protein Isolate	0	0	25.8	25.8
Protein from Pea Protein 85% (Pure Bulk)	0	0	0	0
Protein from Soy Protein Isolate (Supro 661)	0	0	0	0
Protein from L-Cystine	3	2	0.5	0.5
Protein from WG Wheat Flour	0	0	0	0
Protein from WG Brown Rice Flour	0	0	0	0
**Carbohydrate (g)**	**660**	**660**	**482**	**482**
Carbohydrate from Egg White	0	0	1.6	1.6
Carbohydrate from Pea Protein 85% (Pure Bulk)	0	0	0	0
Carbohydrate from Corn Starch	381	381	227.8	227.8
Carbohydrate from Maltodextrin 10	110	110	66	66
Carbohydrate from Lactose	0	0	35	35
Carbohydrate from Sucrose	9	9	139	139
Carbohydrate from WG Wheat Flour	0	0	0	0
Carbohydrate from WG Rice Flour	0	0	0	0
Carbohydrate from Inulin	9	9	12	12
Carbohydrate from Fructooligosaccharides	0	0	0	0
Carbohydrate from Vitamin Mix V10001	1	2	1	1
**Fat (g)**	**72**	**72**	**153**	**153**
Fat from Beef, Powdered (5013)	0	0	35.9	35.9
Fat from ChickPRO	0	0	4.5	4.5
Fat from Fish Protein Isolate	0	0	0.1	0.1
Fat from Pea Protein 85% (Pure Bulk)	0	0	0	0
Fat from Soy Protein Isolate (Supro 661)	0	0	0	0
Fat from WG Brown Rice Flour	0	0	0	0
Fat from Soybean Oil	70	72	0	0
Fat from Butter, Anhydrous	0	0	30	30
Fat from Canola Oil	0	0	16	16
Fat from Coconut Oil, 76	0	0	0	0
Fat from Corn Oil	0	0	36	36
Fat from Menhaden Oil	0	0	0	0
Fat from Olive Oil	0	0	0	0
Fat from Peanut Oil	0	0	8	8

Diet	Standard CHOW (CH)	Leucine rich CHOW (CH+AA)	American Diet (AD)	Leucine rich AD (AD+AA)
Fat from Primex-Z (Shortening)	0	0	22	22
**Fiber (g)**	**100**	**100**	**33.7**	**33.7**
Fiber from ChickPRO	0	0	1.7	1.7
Fiber from WG Wheat Flour	0	0	0	0
Fiber from WG Rice Flour	0	0	0	0
Fiber from Cellulose	75	75	0	0
Fiber from Inulin	25	25	32	32
Fiber from Fructooligosaccharides	0	0	0	0
**g%**				
**Protein (g%)**	**17**	**17**	**19.5**	**19.5**
Protein from Casein	16	0	3.1	3.1
Protein from Beef, Powdered (5013)	0	0	6.2	6.2
Protein from ChickPRO	0	0	5.4	5.4
Protein from Egg White, Dried	0	0	1.9	1.9
Protein from Fish Protein Isolate	0	0	2.9	2.9
Protein from Pea Protein 85% (Pure Bulk)	0	0	0	0
Protein from Soy Protein Isolate (Supro 661)	0	0	0	0
Protein from L-Cystine	0	0	0.1	0.1
Protein from WG Wheat Flour	0	0	0	0
Protein from WG Brown Rice Flour	0	0	0	0
**Carbohydrate (g%)**	**62**	**63**	**54.2**	**54.2**
Carbohydrate from Egg White	0	0	0.2	0.2
Carbohydrate from Pea Protein 85% (Pure Bulk)	0	0	0	0
Carbohydrate from Corn Starch	36	36	25.6	25.6
Carbohydrate from Maltodextrin 10	10	11	7.4	7.4
Carbohydrate from Lactose	0	0	3.9	3.9
Carbohydrate from Sucrose	1	1	15.6	15.6
Carbohydrate from WG Wheat Flour	0	0	0	0
Carbohydrate from WG Brown Rice Flour	0	0	0	0
Carbohydrate from Inulin	1	1	1.3	1.3
Carbohydrate from Fructooligosaccharides	0	0	0	0
Carbohydrate from Vitamin Mix V10001	0	0	0.1	0.1
**Fat (g%)**	**7**	**7**	**17.2**	**17.2**
Fat from Beef, Powdered (5013)	0	0	4	4
Fat from ChickPRO	0	0	0.5	0.5

**Diet**	**Standard CHOW (CH)**	**Leucine rich CHOW (CH+AA)**	**American Diet (AD)**	**Leucine rich AD (AD+AA)**
Fat from Fish Protein Isolate	0	0	0	0
Fat from Pea Protein 85% (Pure Bulk)	0	0	0	0
Fat from Soy Protein Isolate (Supro 661)	0	0	0	0
Fat from WG Brown Rice Flour	0	0	0	0
Fat from Soybean Oil	7	7	0	0
Fat from Butter, Anhydrous	0	0	3.4	3.4
Fat from Canola Oil	0	0	1.8	1.8
Fat from Coconut Oil, 76	0	0	0	0
Fat from Corn Oil	0	0	4	4
Fat from Menhaden Oil	0	0	0	0
Fat from Olive Oil	0	0	0	0
Fat from Peanut Oil	0	0	0.9	0.9
Fat from Primex-Z (Shortening)	0	0	2.5	2.5
**Fiber (g%)**	**9**	**10**	**3.8**	**3.8**
Fiber from ChickPRO	0	0	0.2	0.2
Fiber from WG Wheat Flour	0	0	0	0
Fiber from WG Rice Flour	0	0	0	0
Fiber from Cellulose	7	7	0	0
Fiber from Inulin	2	2	3.6	3.6
Fiber from Fructooligosaccharides	0	0	0	0
**Protein (kcal)**	**708**	**708**	**695**	**695**
Protein from Casein	696	0	111	111
Protein from Beef, Powdered (5013)	0	0	219	219
Protein from ChickPRO	0	0	191	191
Protein from Egg White, Dried	0	0	68	68
Protein from Fish Protein Isolate	0	0	103	103
Protein from Pea Protein 85% (Pure Bulk)	0	0	0	0
Protein from Soy Protein Isolate (Supro 661)	0	0	0	0
Protein from L-Cystine	12	9	2	2
Protein from WG Wheat Flour	0	0	0	0
Protein from WG Brown Rice Flour	0	0	0	0
Carbohydrate (kcal)	2642	2642	1930	1930
Carbohydrate from Egg White	0	0	7	7
Carbohydrate from Pea Protein 85% (Pure Bulk)	0	0	0	0
Carbohydrate from Corn Starch	1524	1524	911	911

**Diet**	**Standard CHOW (CH)**	**Leucine rich CHOW (CH+AA)**	**American Diet (AD)**	**Leucine rich AD (AD+AA)**
Carbohydrate from Maltodextrin 10	440	440	264	264
Carbohydrate from Lactose	0	0	140	140
Carbohydrate from Sucrose	36	34	556	556
Carbohydrate from WG Wheat Flour	0	0	0	0
Carbohydrate from WG Rice Flour	0	0	0	0
Carbohydrate from Inulin	38	38	48	48
Carbohydrate from Fructooligosaccharides	0	0	0	0
Carbohydrate from Vitamin Mix V10001	4	6	4	4
**Fat (kcal)**	**652**	**652**	**1376**	**1376**
Fat from Beef, Powdered (5013)	0	0	323	323
Fat from ChickPRO	0	0	40	40
Fat from Fish Protein Isolate	0	0	1	1
Fat from Pea Protein 85% (Pure Bulk)	0	0	0	0
Fat from Soy Protein Isolate (Supro 661)	0	0	0	0
Fat from WG Brown Rice Flour	0	0	0	0
Fat from Soybean Oil	630	652	0	0
Fat from Butter, Anhydrous	0	0	270	270
Fat from Canola Oil	0	0	144	144
Fat from Coconut Oil, 76	0	0	0	0
Fat from Corn Oil	0	0	324	324
Fat from Menhaden Oil	0	0	0	0
Fat from Olive Oil	0	0	0	0
Fat from Peanut Oil	0	0	72	72
Fat from Primex-Z (Shortening)	0	0	198	198
**Total (kcal)**	**4001**	**4001**	**4000**	**4000**
**kcal/g**	**3.7**	**3.8**	**4.5**	**4.5**
**kcal%**				
**Protein (kcal%)**	**18**	**18**	**17**	**17**
Protein from Casein	17	0	3	3
Protein from Beef, Powdered (5013)	0	0	5	5
Protein from ChickPRO	0	0	5	5
Protein from Egg White, Dried	0	0	2	2
Protein from Fish Protein Isolate	0	0	3	3
Protein from Pea Protein 85% (Pure Bulk)	0	0	0	0
Protein from Soy Protein Isolate (Supro 661)	0	0	0	0

Diet	Standard CHOW (CH)	Leucine rich CHOW (CH+AA)	American Diet (AD)	Leucine rich AD (AD+AA)
Protein from L-Cystine	0	0	0	0
Protein from WG Wheat Flour	0	0	0	0
Protein from WG Brown Rice Flour	0	0	0	0
**Carbohydrate (kcal%)**	**66**	**66**	**48**	**48**
Carbohydrate from Egg White	0	0	0	0
Carbohydrate from Pea Protein 85% (Pure Bulk)	0	0	0	0
Carbohydrate from Corn Starch	38	38	23	23
Carbohydrate from Maltodextrin 10	11	11	7	7
Carbohydrate from Lactose	0	0	3	3
Carbohydrate from Sucrose	1	1	14	14
Carbohydrate from WG Wheat Flour	0	0	0	0
Carbohydrate from WG Rice Flour	0	0	0	0
Carbohydrate from Inulin	1	1	1	1
Carbohydrate from Fructooligosaccharides	0	0	0	0
Carbohydrate from Vitamin Mix V10001	0	0	0	0
**Fat (kcal%)**	**16**	**16**	**34**	**34**
Fat from Beef, Powdered (5013)	0	0	8	8
Fat from ChickPRO	0	0	1	1
Fat from Fish Protein Isolate	0	0	0	0
Fat from Pea Protein 85% (Pure Bulk)	0	0	0	0
Fat from Soy Protein Isolate (Supro 661)	0	0	0	0
Fat from WG Brown Rice Flour	0	0	0	0
Fat from Soybean Oil	16	16	0	0
Fat from Butter, Anhydrous	0	0	7	7
Fat from Canola Oil	0	0	4	4
Fat from Coconut Oil, 76	0	0	0	0
Fat from Corn Oil	0	0	8	8
Fat from Menhaden Oil	0	0	0	0
Fat from Olive Oil	0	0	0	0
Fat from Peanut Oil	0	0	2	2
Fat from Primex-Z (Shortening)	0	0	5	5
**Saturated Fat (SFA, kcal%)**	**2**	**2**	**12**	**12**
**Monounsaturated Fat (MUFA, kcal%)**	**4**	**4**	**13**	**13**
**Polyunsaturated Fat (PUFA, kcal%)**	**9**	**10**	**8**	**8**
**Total Fiber (g/1000 kcal)**	**25**	**25**	**8.4**	**8.4**
